# "The Hospital" Nursing Mirror

**Published:** 1898-01-22

**Authors:** 


"The Hospital" Nursing Mirror.

Being the Nursing Section of "The Hospital."

[Contributions for this Section of "The Hospital" should be addressed to the Editor, The Hospital, 28 & 29, Southampton Street, Strand, London, W.C., and should have the word "Nursing" plainly written in left-hand top corner of the envelope.]

### News from the Nursing World.

### THE QUEEN AND MISS SUSAN CATOR.

Yet another nurse has been awarded, by command of Her Majesty, the order of the Royal Red Cross for services to the sick and wounded. Miss Susan Cator is the name of the latest recipient of this much-coveted honour.

### THE DUCHESS OF FIFE AND THE SICK CHILDREN.

On Tuesday, the 11th inst., the Duke and Duchess of Fife visited the Hospital for Sick Children at Great Ormond Street, and 140 little ones had the honour and pleasure of receiving their toys from the Duchess's own hands.

### NURSES WANTED FOR INDIA.

The Viceroy of India telegraphs for twenty-five more nurses, two more lady doctors, and eight doctors; and if the plague continues more will be wanted. Nurses who wish to volunteer must send in their names to the Revenue Secretary, India Office, Whitehall, S.W., and they will receive full information. None but those possessing full qualifications and who are found by the Medical Board to be fit for tropical service will be accepted. The first party will be chosen from those whose names are entered as ready to start at a few days' notice, and the names of those applying now will be placed on the list of volunteers ready to start at the next call.

### PUERPERAL FEVER IN ACHILL ISLAND.

We have received information as to the prevalence of puerperal fever in Achill Island which makes one wonder whether the good tidings of antiseptic midwifery has yet reached the inhabitants of that distant isle. It is precisely in poor and outlying districts that bad methods become traditional among mothers and the wise women who nurse them in their confinements, and few things are more difficult than to alter the habits of ignorant and prejudiced people. Still, if the doctor and the nurse will conspire to preach, in season and out of season, the great doctrine of cleanliness and antiseptics, and if some good people will furnish better supplies of clean linen than are often to be found in outlying districts, puerperal fever ought to be abolished even in the wilds of Achill.

### MR. ERNEST HART AND LADY DOCTORS.

It must afford lady doctors considerable satisfaction to know that such a strong and clear intellect as Mr. Ernest Hart's countenanced their efforts. For several years he and Mrs. Hart gave a scholarship at the London Woman's School of Medicine. He did not always take a favourable view of the movement, and Mrs. Garrett Anderson, M.D., surmises that possibly Mrs. Hart, who studied medicine in Paris, might have had something to do with his change of opinion.

### NURSING SETTLEMENT AT PLAISTOW.

The following resolution was passed at a meeting of medical men of East Ham and district to consider the proposed scheme of the "Plaistow Maternity Charity and District Nurses' Home" (Sister Katherine's) to establish a permanent nurses' settlement in East Ham: "We, the undersigned, agree that, considering the nurses do now attend midwifery cases who can well afford to pay the ordinary medical fees (instances were given bearing out this assertion by several men present), it is superfluous, and a great abuse of the moneys of the charitable public, to further enlarge this so-called charity, as now carried on, to the detriment of the many qualified medical men in the neighbourhood." Here followed the signatures of 52 medical men—practically the whole of the medical profession of East Ham and district.

### THE DISGUYSED MINSTRELS AT GUY'S HOSPITAL.

The Disguysed Minstrels' Club gave a capital entertainment at Guy's Hospital on Friday, January 14th. The programme consisted partly of negro and comic songs, and partly of the new and original tragedy of "Ben Trovato; or, The Pursuit of the Well-beloved," which was delightfully funny. The "Twin Duet," too, was most amusing.

### THE QUEEN VICTORIA CITY OF LINCOLN NURSING FUND.

The Queen has graciously given permission that the nursing fund of Lincoln, raised to commemorate this auspicious year, shall bear the name of the Queen Victoria City of Lincoln Nursing Fund. The capital for investment, it is hoped, will reach the handsome sum of £5,000, to which Miss Bromhead has added £163 from the funds of the Bromhead Institute.

### NURSES FOR BANGKOK.

Two nurses will start for Bangkok, in Siam, this month. They have been engaged by the Colonial Nursing Association.

### TYPHOID AT EXETER.

We are sorry to hear that owing, as is presumed, to the insanitary condition of the Bowring Ward of the Devon and Exeter Hospital, from which the patients have now been removed, three nurses were stricken with typhoid fever. One, Nurse Hallett, a young nurse, has unfortunately died, and the others are in a precarious state. This shows how urgent has been the need of the recently occupied new wing. We understand the committee will not resume the use of the Bowring Ward for the children.

### TEMPORARY QUARTERS.

The immediate effect of the grant from the Prince's Fund towards the opening of the closed wards at Guy's Hospital is that a portion of the nursing staff whose sleeping accommodation was arranged in them for the time being, have been provided with a temporary lodging without the gates, until the new nurses' home in course of erection is ready for occupation.

### THE YOUNG HELPERS' LEAGUE.

On Saturday last, at the Royal Albert Hall, the "Young Helpers' League" gave their sixth annual entertainment in aid of funds to support the numerous unfortunate waifs that drift into Dr. Barnardo's Homes who are incapable of earning their own living in consequence of some physical defect. These young folk assembled from the different institutions belonging to Dr. Barnardo's Homes, and entered into the games and other items of the programme with great zest; whilst the Duchess of Somerset kindly crowned the victors, and received purses on behalf of the charity.

### LONDON TEMPERANCE HOSPITAL.

THE following courses of lectures have been arranged for this year for the nurses at the London Temperance Hospital: Twelve lectures on "Hygiene as Applied to Nursing," by the late Sir B. W. Richardson; twelve on "General Anatomy and Physiology as Applied to Nursing," by W. J. Collins, M.D., M.S., F.R.C.S.; a course of lectures on "The Theory and Practice of Nursing," by Miss Orme, the lady superintendent; and "Lessons in Dispensing," by Miss De Lassasie, the lady dispenser. Only paying probationers are taken at this hospital, and the age preferred is from twenty-five to thirty. A certificate is granted at the end of each year's satisfactory work. A nurse probationer's certificate is given to probationers at the conclusion of one year's work if desired. Probationers at the end of two years' satisfactory work receive a bronze medal, a certificate stating their length of training, and are entitled to be called "nurse"; whilst nurses completing their three years' training may compete for the gold medal which is awarded yearly.

### SISTER MONICA.

THE Inspector of Queen Victoria's Jubilee Nurses' Institute, Miss Peter, very kindly informs us that Sister Monica is midwife-nurse to a lying-in charity, and is not connected with the Warwick affiliated branch of Q.V.J.I.

### LADY ROBERTS' NURSES.

THREE of Lady Roberts' nurses have been lent for temporary duty to the British General Hospital at West Ridge, Rawal-pindi. They are under the orders of the Surgeon-General commanding the British troops in India.

### THE LADY INSPECTOR OF BOARDED-OUT CHILDREN.

THE Committee of the Howard Association, London, have placed on record a resolution, supporting Miss Mason, H.M. Inspector of Boarded-out Children, who has recently been subjected to severe criticism by her fellow workers. Miss Mason has, in their opinion, been indefatigably vigilant in her work, and has rendered important service to the State, and to the children under her care.

### ARGYLLSHIRE DISTRICT NURSING ASSOCIATION.

THE Argyllshire District Nursing Association, which was founded in October last by the Duchess of Argyll, has lost no time in setting to work. At its first meeting it was resolved to establish a nurse in each of the four districts of the shire. The nurse established at Easdale, in the district of Lorn, has already seen much service, and done excellent work during the recent outbreak of typhoid; another is busy at Stralachlan, in the district of Cowal; another has been secured to work in the Mid-Argyll district of Ardrishaig and Lochgilphead; and still another is for Kintyre, to be stationed at Tarbert. As appetite grows with eating, so work increases with the doing of it; and it is now understood that the inaugurators of the association will not rest content until every district in Argyll has its Queen's nurse. This is a healthy and happy ambition, and we trust that it will be realised in the near future.

### A CHILDREN'S TREAT.

THE superintendent and nurses of the Middlesborough Nursing Association recently entertained 500 poor children to tea. Lady Zetland is a cordial supporter of this annual entertainment, and the children each received a toy from her hands. Nurses in hospital make Christmas bright for their patients, and the idea seems spreading to nursing associations. It is a very happy one, especially if, as in this case, the leading citizens and tradesmen unite in providing the necessary money.

### HULL'S JUBILEE NURSES' HOME.

THE minutes of the Nurses' Home Sub-Committee showed that the sum of over £3,000 had been collected, and at the meeting of the Jubilee Celebration Committee on the 12th inst. a resolution, unanimously carried, provided that the money should be spent in building and furnishing a suitable home for eight nurses, and that any remaining balance should be invested as a reserve fund. A tablet will be placed in the new Home announcing that it was erected in memory of Her Majesty's Diamond Jubilee. At present the subscription list produces about £200 a year, and supports two or three nurses. Efforts will be made to bring it up to an adequate amount for the proposed increase of staff.

### SHORT ITEMS.

THE annual meeting of the Buxton District Nursing Association was held in the Devonshire Hospital, when it was stated that the income of the association amounted to £132, and that the balance in hand was £10. The Duke of Devonshire was elected president. —The nurses and medical staff of the Royal Infirmary, Windsor, gave a dramatic entertainment to the inmates, which was very successful.—Sister Helen has begun her nursing classes on Wednesday afternoons at Hythe. The fee for the course of ten is only 2s. 6d., and so far they have been well attended.—The floors in some of the wards at the London Temperance Hospital are of adamantine mosaic, which is found to be much less tiring to the nurses' feet than those of polished wood.—Mr. Walker presided over the annual meeting of the Bungay Nursing Association. Unfortunately, Nurse Allinson retired owing to ill-health, and her successor, Nurse Prentice, only stayed six months, after which Nurse Nathan was appointed. These changes caused a heavier outlay than usual. The work of the nurse is about twelve visits a day.—Eight lectures on Sick Nursing will be given in the Lecture Hall of the Technical School, Maudesley Street, Bolton, by Mrs. Tobitt, lecturer to the Lancashire County Council. The first lecture will be free, and the cost of the course is only 1s. 6d.—The medical and nursing staffs of the Marylebone Infirmary have sent a handsome contribution towards the funds of the Church Army. The Church Army mission nurses receive their training at the infirmary.—A sum of £145 and a subscription of £10 was announced at the close of a meeting which unanimously resolved to establish a district nurse in the Crieff District, Scotland.—Upwards of £500 has been subscribed towards the endowment of a bed in the Great Northern Central Hospital in memory of H.R.H. Princess Mary Duchess of Teck.

### Practical Aspects of a Nurse's Life.

By Sister Grace.

XIV.—SISTERS (*concluded*).

In surgical wards the operative work presses somewhat heavily on the sister, because it absorbs all her energies of body and mind and keeps her always in a state of high tension, and this state of things is of necessity never ending, the moment does not arrive when she may take breath and rest from anxiety for a few days; some among the patients are always hovering on the borders of the valley of the shadow of death.

Theatre work varies much according to the management, and the degree of consideration shown by the surgeons, but under the most favourable circumstances a sister engaged in it must always lead a life of anxiety. Endeavour to cultivate a calm demeanour; a sister who is never hurried or fussy gives confidence to all who work with her, and naturally in operation and accident wards there is plentiful opportunity of cultivating such gifts. The arrival of one or more severe accidents is apt to cause young nurses to what they call "lose their head," but when they see their sister, as calm and unexcited as usual, giving her orders without hurry or confusion, they become reassured and soon learn to imitate what they admire; we are but creatures of imitation, and probationers endeavour to tread in the footsteps of their sister.

Remember in operation work never to run the risk of being weighed in the balance and found wanting. It takes some practice to have at hand everything that the most fertile surgical brain could conceive as necessary to each operation; all surgeons have their own favourite methods of procedure and their own favourite dressings—never forget them, but have also others in case they should seek a new departure. Dressings are far simpler of late years; when I began nursing we were in the thraldom of the dressings requiring an elaborate arrangement of a certain arbitrary number of thicknesses of gauze and pink macintosh, and I remember, for some surgeons, being obliged to sew them neatly in place. Now things are easier, and we are not teased by the presence of the spray, which made an unpleasant fizzing noise and covered everyone near with a fine dew like the result of a winter fog.

Duties to Probationers.

Whatever the custom prevailing in your hospital with regard to lectures and the professional education of the nurses, it is certainly one of the most serious and important duties of a sister to instruct those under her in all the details of their work. It is not, perhaps, the pleasantest part of your work, especially if teaching is repugnant to you, as it is to many; but the appreciation generally shown by the recipients of your lessons will amply repay you, and one must always feel a real pride in turning out thoroughly good and capable nurses.

Remember, in dealing with those under you, that once you too were a beginner, and try to remember how stupid, how unobservant, and how slow you were. We are, perhaps, all of us a little too much inclined to forget those past days. Try to make them feel that you are their best friend, and though never relaxing discipline, let them be assured of your sympathy, kindness, and justice in all dealings with them.

Nurses never mind a sister being very strict, and even, perhaps, a little severe, if she is at the same time absolutely just and strictly impartial, and if they feel that she has their real interests at heart. Never find fault when you are angry; let a little time elapse, so that your irritation may have died down and you have well reflected on the subject; this has a good moral effect—first, because the culprit feels that you are not one to speak hastily; and, secondly, because the sense of the deliberation with which Nemesis is overtaking her has a very alarming effect, and one less easily forgotten than the impression left by the utterance of a few hasty, ill-considered words.

Again, when you have had occasion to blame a nurse, perhaps seriously, do not remember it against her; treat her as before, unless she should again appear unworthy; do not give her the disheartening feeling that she can never redeem herself in your eyes.

Never "nag" at your nurses, if you will forgive the homely phrase. Speak to the point, and then let it rest; no good is ever gained by making a horse fret against the curb.

You will do more by the force of your own example than by any amount of blame and fault-finding; let all see that your duty comes before everything, and if you are strict with others you are still more strict with yourself. Never be a single minute late on duty, and you will find that you need not complain of want of punctuality in others. Be extremely respectful and attentive to all the house surgeons, upholding their authority before patients and nurses, and you will never have cause to complain of flippant behaviour in your probationers. Be at all times ready to give up your own convenience and leisure for the sake of others, and you will not often be vexed by selfishness amongst your subordinates.

With regard to your patients, act on the same lines; do not fret them with numberless futile rules and regulations, but see that the few you make are absolutely obeyed. Never raise your voice irritably or speak without due consideration, but try to bring all under your care to such a state of respect for you and for your wishes that a look of warning shall be sufficient.

I have myself always had male patients, who, I fancy (perhaps without reason), are more easily managed than women, but different difficulties are to be encountered in each case, and you must learn to overcome them. Sometimes a discreet short-sightedness is useful, and to look the other way is wise if no rule is being broken. Your word must be law, but do not make the laws too numerous. In conclusion, let me say that it is a happy life, for it is one where you have little time to think of yourself. To make a good sister you must give up everything else, and always be at hand to rule your little kingdom, and to dispense comfort and cheerfulness therein. "Love yourself last," must be your watchword, and you will I hope, when the time comes for you to give up your sceptre, feel that the years spent within that narrow kingdom have been very happy and blessed ones.

### Wants and Workers.

[The attention of correspondents is directed to the fact that "Helps in Sickness and to Health" (Scientific Press, 28 & 29, Southampton Street, Strand, London, W.C.) will enable them promptly to find the most suitable accommodation for difficult or special cases.]

Nurse Longstaffe, 61, Divinity Road, Cowley St. John, Oxford, wishes to know if there is any nurse past work who would like to live in Oxford with one who takes private cases, and who would keep house in return for board and lodging.

E. M. Chenery, 29, Listria Park, Stoke Newington, would be glad to know of a home by the sea (South Coast) where a nurse suffering from the effects of over work can go on reduced terms. See answer in "Notes and Queries" this week.

Could anybody help by giving a second-hand mail-cart for a little paralysed boy, age ten years old, so that he may be able to be sent to school? The school is some distance from his home, and he is unable to walk so far. If anybody is kindly willing to help in this matter, kindly communicate to The Nurse, The Home, Long Ashton, near Bristol.

Mary D. Marshall, Wallingford, Bucks, asks if anyone can tell her of an institution in England where an idiot boy of seven years of age who is also blind can be received.

Could you kindly tell me of any home where a young child (boy) who has epileptic fits and is partially imbecile could be received? The father (who is a working man) might be able to pay a small sum towards his maintenance.—Matron, Rous Memorial Hospital, Newmarket.

### Post-Graduate Clinics for Nurses.

### BED SORES: THEIR PREVENTION AND CURE.

GENERALLY speaking, bed sores are the result of neglect or inefficient nursing. There are, however, certain conditions in which it is almost impossible to avoid their occurrence, even with the greatest care—in myelitis, for instance, where bed sores may be considered, practically speaking, as a complication of the disease. In most diseases due to spinal lesions or injuries, and in Bright's disease, there are trophic changes which lower the vitality of the tissues and render them peculiarly susceptible to injury. In nursing such cases this fact should not be forgotten, but should lead to even more than ordinary care in the avoidance of bed sores. Pressure, the irritation of urine or other moisture, creases in the under sheet and blanket or mackintosh, and crumbs in the bed, are their chief causes. The pressure of hard, dry crumbs in the bed, though giving rise to much discomfort, does not seem at first sight to be likely to cause bed sores. I have seen, however, more than one such case, where the hard crumb made the first abrasion in the skin of a paralysed patient, and where, owing to the hyperæsthesia due to his disease, the discomfort caused by the crumb was magnified almost to torture.

Prevention is, therefore, the nurse's chief care. Pressure must as far as possible be avoided, and especially continued pressure, over prominent bones, the sacrum, trochanters, knees, ankles and elbows. This can be attained by protection of prominent parts from contact with a hard surface, and as frequent a change of position as the nature of the case will allow. In cases of paralysis a water pillow is an absolute necessity, where a complete water bed cannot be obtained. The judicious use of pads, in the form of rings resembling a miniature lifebuoy, will do much to relieve pressure. They can be made by twisting sufficient tow into a ring, wrapping it round with common cotton-wool, and winding it over and over with a flannel or cotton bandage. These rings should, without being hard, be sufficiently firm not to go flat under the patient's weight and so allow the part they are designed to protect, to touch the bed. They are of great use in guarding the bones of emaciated persons or to place beneath the sacrum of a patient where a Bryant or other splint necessitates his lying on his back. Where, however, there is great loss of vitality in the tissues, the pads may only relieve pressure at one point at the expense of the surrounding parts becoming in themselves a source of danger.

Change of position is very important. Many patients have a habit of always lying on one side; they should be encouraged to turn from time to time, lying for a few hours on either side, keeping the favourite position to begin the night, as otherwise sleep may be interfered with. The elbow, knees, and above all the heels and ankles, must not be neglected; heel sores are only too frequent, especially in paralysis. In such cases the heels should never be allowed to rest on the bed, but should be raised from it by a rolled drawn sheet or a pad placed lengthwise under the ankles across the bed. This pad should be carefully kept in place. The heels should be washed night and morning with methylated spirit and dusted with zinc or starch powder. The elbows and knees may also require this attention. Sometimes it is necessary to protect these joints by wrapping them in cotton wool and a flannel bandage.

### CLEANLINESS AND CARE OF THE SKIN.

The back and hips should be washed, rubbed with methylated spirit or spirits of wine at least once a day and powdered with zinc or starch powder. Where there is moisture a sort of cream may be made in the palm of the hand with olive oil and zinc powder, and rubbed gently all over the parts exposed to it, or olive oil alone may be used. With harsh dry skin it is often better to use oil than powder. I have nursed several cases of perineal section, vesico-vaginal fistula, and other cases of incontinence of urine, or fæces, and found a liberal application of oil most valuable in preventing the irritation and excoriation of the skin and resulting bed sores.

Where special urinals cannot be had or do not succeed, the absorbent pads described by your correspondent are most useful. Of course they must be removed and burnt at once when wet, for in careless hands they are a source of dirt and danger, acting as a most offensive poultice. Whenever the sheet is wet it must be "drawn," and the patient's back dried and oiled, or washed if necessary. The inside of the thighs must not be forgotten.

To cure a bed sore is far more difficult than to prevent one forming. As soon as the skin shows signs of breaking, painting the threatened spot with colodion flexile may prevent further mischief, and, of course, the patient must not lie on that side. When the skin is broken, a piece of lint large enough to allow at least half an inch margin all round the sore should be spread with boracic or zinc ointment or Lassar's paste, and fastened over the wound with a piece of strapping cut still larger and snipped at the edge to give it a firm hold. Great care must be taken that the dressing does not slip and rub the injured surface. In bed sores where there are sloughs, these are best removed by the aid of hot fomentations and the use of iodoform. The wound may be advantageously syringed with Jeyes' fluid diluted in warm water. When the sloughs come away, if the wound looks sluggish, granulation may be encouraged by packing with a small piece of lint soaked in red lotion, care being taken to avoid the healthy tissue. Lint soaked in boracic lotion makes a good packing. The whole of the injured surface should be covered with boracic ointment spread on lint and fastened with broad strips or circular pieces of strapping as described above.

Where the dressing of bed sores is left to the discretion of the nurse, she may find the above suggestions of use; but, of course, the doctor in charge of the case may have methods of his own, and other ways may be learnt as good or far better, both for their prevention and, where possible, their cure.

### The Workhouse Nurse.

AN important letter appears in the *Times* for the 18th inst., signed by the chairman of the Northern Workhouse Nursing Association explaining the policy and successful working of this nursing association and the causes that render it so difficult for guardians to get nurses for their workhouses. For those in some of the big centres there are lists of candidates waiting "dozens deep." It is in the small unions that the difficulty exists, and in the writer's opinion it is because the conditions of work are adverse. In the first place "those who want a good thing must pay for it," and a salary of £20 a year, with board, lodging, and washing, is not enough to get a good nurse, and he thinks that "caps, aprons, collars, and cuffs" ought to be included in the term "uniform." He says, "It would be just as reasonable to tell Tommy Atkins that, in giving him a tunic alone, you have provided him with a uniform, as it would be to consider a nurse's dress full uniform." The second drawback is the minority of "meddling" matrons still remaining; and the last cause, but by no means least, is that the diet and holidays are as a rule insufficient. The food is rarely varied, and is often badly cooked, whilst the quantity of nitrogenous food is, as regards breakfasts, decidedly deficient.

### Nursing in South Africa.

(From the *South African Medical Journal*. By WALTER HARRIS, M.R.C.S.)

A NEW departure, and one of great importance as regards the nursing at the Provincial Hospital, Port Elizabeth, has recently been inaugurated. A matron has been appointed, and a commodious Nurses' Home is shortly to be built, the plans being already drawn and the ground selected.

That the third largest hospital in the colony should not have been long ago provided with a matron may seem a matter of surprise; but the building is the key to the appointment, in that there has absolutely been no room to house any but actual workers in the wards, and the duties of these have been so onerous that the fact of the work having been done at all has been ample proof that it has been faithfully performed. Without increase of members, and especially of juniors, a matron would have found no occupation, and such an appointment would not have aided the already hard-driven staff.

To show how utterly inadequate the number of nurses has been to the work, it needs but to say that, including those in the children's ward, charge, staff, and junior nurses have totalled but nine women. These had to take the care of all patients, excepting coloured men and alcoholic or venereal whites. When one notes that there were in 1896 more Europeans treated in the Provincial Hospital than in the hospital at Kimberley, with its staff of 40 nurses and probationers, the situation becomes absurd to all but the workers, whose position has frequently been thankless and one of hardship.

For a very long time past the medical staff have advocated the building of suitable quarters for the nurses, but the cry of economy has been raised. One member of the board of managers went so far as grimly to propose that the weekly visitors should be paid to keep away, in case they might recommend the spending of any money.

How unsuitable has been the residential provision for nurses may be illustrated by the fact that those on night duty have had in the daytime to sleep, or try to, almost immediately over the kitchen, with every sound therefrom and tradesmen's carts scraping the gravel under their windows penetrating to their rooms, while those on day duty have been afraid to speak at meals, lest they should wake the sleepers above, where five small rooms constituted their joint dormitories.

In consequence, however, of my plain words in the report of the medical staff for last year, steps were at length taken to erect a building large enough to accommodate a matron and twenty nurses, and this is now at the point of commencement.

In the meanwhile changes in the medical staff have provided a little more space, for, when the resident surgeon resigned at the end of last September, the board appointed a house surgeon in his stead, increasing the visiting staff, and giving into their hands the principal control of patients. At the same time they have put the bachelor house surgeon back into the old quarters in the main buildings, thus leaving free the residence built for the resident surgeon in 1891. In this house *pro tem.* the matron is lodged, with some of the nurses. This arrangement is obviously but a temporary one, as there will be ample room for all in the new nurses' building. It is assumed by the public that the surgeon's house will then be used to receive private patients. This, indeed, seems the fairest alternative under the circumstances, as it was built with Government aid on the express understanding that by the surgeon's removal from the hospital proper, the vacated rooms would be available to meet the increased demand for private wards, and now these have again been requisitioned for surgeon's quarters. This backward move in the executive to the condition of 20 years ago need not, however, further concern us in this section.

The board of managers of the Provincial Hospital must certainly be congratulated on their resolve—albeit a tardy one—to bring up their staff to reasonable dimensions, for they have now decided to increase the number of the nurses to 14, plus the matron. These numbers, too, are exclusive of the children's ward nurses, who remain under the control of the St. Peter's Sisters, who have held this charge since the opening of the ward in 1888, and whose term has yet several years to run. This will give time to ensure that their very excellent record is ultimately given over into hands tried and proved in the general nursing of the institution.

For the post of matron there were many applications before the board from ladies with most excellent credentials, some of the candidates having had both English and colonial experience in a similar capacity. The choice fell on Mrs. E. E. Fuller, then matron of the Vryburg Hospital, and we will not doubt that she will fully justify the preference. The task before her is, however, an onerous one, and will require the exercise of most of the cardinal virtues to carry to such an issue as the public expect and anticipate. We observe on reference to the official register that Mrs. Fuller was trained as a nurse in Kimberley Hospital, and passed the Government qualifying examination in June, 1895. Her experience is short, but not incompatible with success, though the fact that she must often in the future have control of nurses of greater seniority will doubtless render the position more difficult. At the same time, the merit will be greater if success be attained.

The principal need of the hospital has been, and is, the training of probationers. On this point great stress was laid at the board when it was decided that a matron be appointed. We cannot, therefore, doubt that a primary weight was given to this qualification of the matron when the appointment was made. The need is great to institute a proper training school at so important a centre as Port Elizabeth. The staff who up to now have so ably performed the nursing duties of the hospital, in spite of difficulties, are too conversant with their work to require instruction, and if in the future their record is equalled, it will be well; for perfectly trained and long experienced nurses from some of the first schools in the world have had charge of its wards. The special requirement is to create a creditable school locally, and help utilise some of the hundreds of well-informed and brought-up young women who are constantly volunteering as probationers. The two or three nurses who have qualified from the hospital of late years have been individually instructed by the medical officers and senior nurses, but to the matron's share should fall the principal work of training, comprising the delivery of lectures and a thorough grounding of juniors in the elements of practical work.

All this will doubtless mean extra expenditure, but we have no doubt funds will be forthcoming for the purpose, as well as to supply the many deficiencies in the institution which a false economy has allowed to exist. Although the hospital is largely and generously supported by the town and province generally, it yet remains the fact that the Government subsidy stands at the same figure as when half the number of patients were received, and is far less in proportion to the work done than in any other State-aided hospital in the colony.

We trust in the near future to see at the Provincial Hospital the weight of the ward work borne by more shoulders, to the content both of patients and nurses. Also a school established, capable of preparing many colonial girls, not only for the examination of the Medical Council, but to become really good nurses, alike fit for the duties of their *Alma Mater* and to supply the growing demand for nurses in South Africa.

### In a Leper Hospital.

THE following short note from a nurse in the Coast Hospital, Little Bay, Sydney, will show some of the difficulties which are met with in carrying out any systematic treatment of this terrible disease when it occurs, as it so commonly does, among people who are steeped in ignorance and prejudice :—

"The medical treatment of lepers is pretty well confined to such as the lepers themselves may fancy. As in cases of chronic ulceration the leprous sores and blotches are continually healing or disappearing to break out again in the same place or elsewhere, the medicament used during the temporary betterment they suppose to have been efficacious, and on the relapse the doctor or attendant is accused of tampering with it. Occasionally a slight amputation or bone scraping is performed, but more in deference to their cry that nothing is done for them than from any hope of benefit. Anti-toxins found beneficial in other diseases have been tried with no result. The lepers are recommended to have the parts affected bathed daily with some antiseptic lotion to keep them sweet and clean. To this they generally object. However weak the solution they complain of its strength; it would seem through the skin not being able to act properly. As it drys it contracts, producing a feeling of harshness and discomfort, which may account for their objections. Of drugs, unless self-chosen, they have a distrust, fearing poison; in one instance a leper would not go to bed without a small drug store of one to two dozen pots and bottles handy, as well as brandy and soda, stout, and wine by the bedside as restoratives."

### Royal British Nurses' Association.

A QUARTERLY meeting of the General Council of the Royal British Nurses' Association was held on Friday (the 14th inst.) at the rooms of the London Medical Society, 11, Chandos Street, W., Mr. T. PICKERING PICK in the chair.

After the reports of the Treasurer and the Executive Committee had been adopted,

Dr. BEZLY THORNE moved the following resolution :—

That having regard to the fact that Dr. Bedford Fenwick has formally called the attention of the General Council to certain reasons which led him to resign the office of treasurer, and to the fact that Dr. Fenwick has never laid those reasons in detail before the General Council, either at the time of his resignation or subsequently, the General Council request the Executive Committee to institute an inquiry into the circumstances referred to in a communication made to that body on the 2nd of March, 1894, into the conduct of the business of the Association during the year preceding that date, and for as long a period subsequent to that date as the continuity of events may appear to require.

Miss GRACE GORDON seconded.

Mr. PEARCE GOULD thought that, seeing the circumstances referred to by Dr. Thorne took place four years ago, it would be a pity to carry that resolution, and he therefore moved the following amendment :—

That it is not expedient to request the Executive Committee to make the inquiry suggested by Dr. Bezly Thorne at the present time.

Dr. HERON, in seconding, said that Dr. Bedford Fenwick stood alone on the Council, and he thought it would hardly be a wise thing for the Council to sit in judgment upon a man with whom they had no sympathy.

Mr. E. A. FARDON said that he felt very strongly that the Association was very much indebted to Dr. Bezly Thorne, who had worked very hard, and who had possibly been more misrepresented than any other member of the Association. No one had made greater sacrifices for the Association than Dr. Thorne, and he was entitled to any assistance the Association could give him to enable him to refute the allegations made against him. Still, seeing that the byelaws were at the present time before the Privy Council, he (Mr. Fardon) thought it would be wiser that the Association should confine itself to such business as was absolutely necessary.

Dr. BEZLY THORNE regretted that he could not withdraw his resolution. Years ago he had wanted to bring forward that motion, but had been persuaded not to for reasons of policy. He would accept a postponement of the inquiry, but not the absolute rejection of his motion.

The amendment was put to the meeting, and was carried, 16 voting for it and 5 against. The majority of those present (about 65 in all) abstained from voting.

Dr. BEZLY THORNE announced his intention of withdrawing from the Association, and left the room.

The proceedings shortly afterwards terminated.

### Appointments.

### MATRONS.

UNION INFIRMARY, SUNDERLAND.—Miss Mary Talfourd Buchanan has been appointed as a Charge Nurse in the above infirmary. Miss Buchanan was trained at the Fir Vale Infirmary, Sheffield, and holds the certificate of the London Obstetrical Society.

VICTORIA HOSPITAL, BURNLEY.—Miss Janet Lindsay was appointed Matron of this hospital on the 12th inst. She was trained at the Liverpool Royal Infirmary, and afterwards held the position of sister at that institution, resigning it to become matron of the Kendal Hospital in 1894.

BRITISH LYING-IN HOSPITAL, ENDELL STREET, S.W.—Miss Mabel Cook, who received her training at St. Mary's Hospital, Paddington, and at the Hospital for Women, Soho, was appointed Matron of this hospital on January 12th. Miss Cook was sister of St. Mary's obstetric wards in 1895.

TETBURY COTTAGE HOSPITAL.—Miss Brierley, who was trained at Guy's Hospital, was appointed Matron of this hospital on the 11th inst.

### Minor Appointments.

THE BOROUGH HOSPITAL FOR INFECTIOUS DISEASES, PLYMOUTH.—The Charge Nurse appointed on January 18th is Miss Sarah Winifred Kemp, who was trained at Middlesex Hospital and remained there seven years altogether. She was engaged as charge nurse on the Metropolitan Asylum Board's hospital ships for six months.

THE HEYWOOD DISTRICT NURSING ASSOCIATION, LANCASHIRE.—Miss E. A. Phillips-Taylor has been appointed one of the Nurses of this district. She was trained at the Ulster Hospital for Women and Children, Belfast, where she stayed for more than nine years, after which she was associated for a couple of years with the Manchester Sick Poor Nursing Association.

MONKWEARMOUTH AND SOUTHWICK HOSPITAL, SUNDERLAND.—The Charge Nurse appointed on December 11th is Miss Hephzibah Thompson, who was trained at the North Riding Infirmary, Middlesborough, and was afterwards charge nurse at the same institution.

### BOOKS RECEIVED.

ADAM AND CHARLES BLACK.
"Cairo of To-day." By E. A. Reynolds-Ball, B.A., F.R.G.S.

HODDER AND STOUGHTON.
"A Doctor of the Old School." By Ian Maclaren.

BAILLIÈRE, TINDALL, AND COX.
"Premature Burial: Fact or Fiction." By David Walsh, M.D.
"The Diagnosis of Disease." By J. Porter Parkinson, M.D., M.R.C.P.

THOMPSON AND CAPPER.

"Common-Sense Homœopathy." By J. M. Moore, M.D.

GEO. JONES AND SONS, Birmingham.
"The Origin of Zymotic Diseases." By F. A. Cooper, B.Sc.,Lond.

FANNIN AND CO.
"Manual of Urine Testing." By John Scott, B.A., M.B.

### Everybody's Opinion.

[Correspondence on all subjects is invited, but we cannot in any way be responsible for the opinions expressed by our correspondents. No communication can be entertained if the name and address of the correspondent is not given, as a guarantee of good faith but not necessarily for publication, or unless one side of the paper only is written on.]

### BED SORES.

"S. V. D. N." writes: The result of my big and varied experience of bed sores may be of some use to "M." After carefully washing the back with pure soap (*i.e.*, soap free from soda) and water, apply zinc ointment, and afterwards dust well with boric powder. I have had a heavy patient in bed for two months, with involuntary micturition most of the time; from the first I adopted the above treatment, and she is now perfectly free from sores. Starch powder may be used in place of boric, but a disinfectant is always preferable.

### "A CANCER CASE."

"S.V. District Nurses" write: In answer to District Nurse's enquiries about "pads for cancer patients," we find that square cushions made of old calico or linen and filled with peat moss dust are invaluable. Peat moss is not only absorbent, but also a disinfectant and deodorant. We have made pads of the whole peat moss broken up into small pieces, which answers the same purpose, but the dust is to be preferred. The peat may be had from any dealer in litter for cattle, or from any farmer who may use it for cattle bedding. The price is 1s. 8d. for a large sack, and we find it much cheaper than anything else suitable for the same purpose.

### MENTAL NURSING.

"A Male Nurse-Companion" writes: I am very much interested in The Hospital, and in some of the articles and letters that appear in it from time to time. In my humble opinion the letter of "Male Attendant" in your issue of January 8th is very much to the point as to the duties that are expected in private nursing. I think our duty is first to God; then, if our heart is right, greater care will be taken of the patient, and although the strain is often very heavy to bear, especially when away single-handed, and month after month has to be spent alone. It has fallen more than once to my lot to have my patient's recovery retarded by the interference of anxious relatives, who thought that not enough was being done for him. They endeavour sometimes to undermine the attendant's influence by persuading the patient to act contrary to his wishes. Their only excuse is the terrible sorrow entailed by seeing their dear one in such a sad condition. I am very pleased to see attendants advised to leave alcoholic drinks alone. The head must be kept clear, and how can they persuade their patients to abstain if they indulge themselves?

### THE ROYAL BRITISH NURSES' ASSOCIATION.

Dr. Bezly Thorne writes: The President of the Incorporated Medical Practitioners' Association has written but not answered. I challenged him to substantiate, or withdraw, an allegation which he had made and repeated, in print and in speech, altogether some four times. He has done neither the one nor the other. The first is beyond the power of man. I scarcely anticipated that he would adopt the alternative course, because some nine months ago, on my pointing out to him that his allegation had no foundation in fact, he said that he would inquire into the matter and meet my objections, and to this hour he has not done so. He appeals instead to the members of the Royal British Nurses' Association to accept his deliberate, but unsubstantiated, statement. He may take my word for it that they will not; in the first place, because he has not dared to take up the glove that has been thrown before him, and, in the second, because they know that if I have spoken falsely my words can be disproved, and further that I have always had the courage to tell them the truth. And now I, in turn, appeal to the members of the Royal British Nurses' Association. I invite them to note for their future guidance the fate, when touched by the point of truth, of the rhetorical bubbles which are floated in the air to mislead them. Within the last few weeks I have shown that the retirement in rotation of matrons from the General Council, which has been persistently attributed to official perfidy, was deliberately proposed to the General Council by Dr. Bedford Fenwick, and by him incorporated in the draft of the bye-laws submitted to, and eventually approved by, the Privy Council. I have shown that Dr. Hugh Woods is unable to substantiate his repeated allegation of treachery on the part of negotiators who neither negotiated nor entered into any agreement whatever. Let the members of the corporation ask themselves in the light of such facts why it is that a member of the existing Executive Committee, who is also an ex-president of the Incorporated Practitioners' Association, has thought it necessary to sever his connection with that body? Let them bear in mind that the officers of their choice have not been attacked for the last time, and understand, from the object lessons which I have given them, what are the tactics and what the devices of a faction which has been aptly designated the "small and turbulent."

### BED SORES.

"M. B." writes: I am very sorry to have been the cause of any unkind remarks made about my asking how to prevent bed sores. I have been nursing out in the world since 1886, but have never met with such dreadful bed sores as I found on two patients when I came here. After dressing the sores as I had been taught, they turned to sloughing. It was on account of the sloughing and bad smells that the doctor ordered charcoal. Of course, I know of all the things mentioned in The Hospital. But in a workhouse it is quite impossible to do these. The doctor gets £60 per annum for coming to the infirmary twice a week and finding drugs; and, of course, he will not give more than can be helped. I did not know of this when I wrote to you. I was dreadfully worried, and thought to show your answer to the Board of Guardians, thinking they would then get me the needful things. The charcoal was sent back to be made fine, for truly, if a nurse could do this, there is no time for it. With 27 women and 32 men, two nurses, one night and one day, it makes things very heavy for the day nurse. I have been here three months, and have not had one hour out of these gates, except the first Sunday morning in this New Year, when I went out to church to a place one mile away from here. I do not think I mind what I do, really, if there is only time to do it in. Of course, I find this very different to private nursing. There everything is got to help one to raise the patient; here, if it is wanted, you must pay for it yourself. I have now two paralysis cases, just in; one came with a bed sore, but the other I pray that I may keep from it. I poulticed the one that came in with it, the place being full of pus; it seems to be getting better, and I trust it will. The assistant doctor tried to comfort me by telling me that these aged people had lost all vitality, and it was impossible to cure them. But I fear that I am too soft-hearted for such work as the workhouse infirmary—I mean a poor parish like this. At Brownlow Hill there was plenty of everything for one's use. I thank you very much for your great kindness in the trouble you have taken for me.

*** We are glad to receive "M. B.'s" letter, and think it good that others who have written on this subject should understand how much she has had to put up with. Patients must have the comforts necessary to their condition, and "M. B." should ask the doctor or her committee for what she wants.—Ed. *T. H.*

### Where to Go.

Royal British Nursing Association.—We are requested to state that the final demonstration of the winter course of lectures on Invalid Cooking, by Miss Earle, will be delivered on Tuesday next, January 25th, at 17, Cavendish Square, commencing at 2.30. The subject will be "Diabetic Foods."

### For Reading to the Sick.

"LEAD, KINDLY LIGHT."

**Verses.**

LEAD, kindly Light, amid the encircling gloom,
Lead Thou me on!
The night is dark, and I am far from Home,
Lead Thou me on.
Keep Thou my feet, I do not ask to see
The distant scene, one step enough for me.

I was not ever thus, nor prayed that Thou
Shouldst lead me on.
I loved to choose and see my path, but now
Lead Thou me on.
I loved the garish day and spite of fears
Pride ruled my will; remember not past years.

So long Thy power hath blessed me, sure it still
Will lead me on,
O'er moor and fen, o'er crag and torrent, till
The night is gone;
And with the morn those angel faces smile
Which I have loved long since and lost awhile.
—*J. H. Newman.*

Send out Thy light, for all is dark around me;
I cannot see Thy hand nor hear Thy voice.
Send out Thy light, I weary in this darkness;
Bid Thy poor trembling child with hope rejoice.
Send out Thy light and lead me, Father, lead me,
Beyond this darkness, sorrow, and unrest.
Send out Thy light, and guide me, worn and weary,
To the calm shelter of my Saviour's breast.
—*Clewer Manual.*

Sometimes a light surprises
The Christian while he sings;
It is the Lord, who rises
With healing in His wings.—*J. Newton.*

Where our Banner leads us,
We may safely go,
Where our Chief precedes us,
We may face the foe.
His right arm is o'er us,
He our Guide will be.
Christ has gone before us;
Christian, follow ye!
—*Bernard of Cluny.*

Thine for ever, Thou our Guide,
All our wants by Thee supplied,
All our sins by Thee forgiven,
Lead us, Lord, from earth to heaven.
—*Mary F. Maude.*

**Reading.**

For with Thee is the well of life, and in Thy light shall we see light.—*Psalm* xxxvi. 9.

When we know not what to do, and human counsel fails to relieve our perplexity, then God will inspire us, and if we obey Him in all humility He will not suffer us to go astray. —*S. Francis de Sales.*

It is a good interior practice to make death a light to live by; in other words, doing everything as we shall wish to have done it when we come to die.—*F. W. Faber.*

As many as are led by the Spirit of God, they are the Sons of God.—*Romans* viii. 14.

Everything in the world is in some sort symbolical of some greater, truer existence of the higher world. Christ is the True Light, the True Vine, *i e.*, that which is symbolised in the outer world by the Light and the Vine. In taking symbolical titles to Himself, Christ is not merely adopting to Himself a name which has its primary meaning in some lower phenomenon of the outer world; on the contrary, He is giving us the spiritual key to nature, so that when we see the material object our heart may recognise the spiritual object which it indicates.—*R. M. Benson.*

### Notes and Queries.

**The contents of the Editor's Letter-box have now reached such unwieldy proportions that it has become necessary to establish a hard and fast rule regarding Answers to Correspondents. In future, all questions requiring replies will continue to be answered in this column without any fee. If an answer is required by letter, a fee of half-a-crown must be enclosed with the note containing the enquiry. We are always pleased to help our numerous correspondents to the fullest extent, and we can trust them to sympathise in the overwhelming amount of writing which makes the new rules a necessity. Every communication must be accompanied by the writer's name and address, otherwise it will receive no attention.**

*Continental Nursing.*

(124) I am anxious to know how would be the best way to set to work to hear of an invalid child on the Continent (Paris preferred) who is in want of a nurse with experience in children's nursing. I have now been nursing for nearly 18 months, and have had one year's training at both French and German. If, therefore, I could now find a case on the Continent I could be fitting myself for such a post by studying the language.—*Gertrude Hawley.*

Gertrude Hawley has sketched a very feasible course for herself. She will probably get the work she wants by advertising in, say, the *Morning Post.* Or she might try the *Daily Mail* first; advertisements in that paper are inserted so much more cheaply, and it is widely read.

*Convalescent Home.*

(125) Could you kindly inform me of a convalescent home on South Coast where I could place a relative who is recovering from bronchitis, and now it is only a matter of change to regain her strength? I could pay a little, but not much. Will you give some particulars?—*Nurse H. Martin.*

The Merchant Taylors' Convalescent Home for Ladies, Bognor. Apply E. Nash, The Hall, Threadneedle Street, E.C. Write for form. A doctor's certificate is needed, but the home is free, and travelling expenses from London allowed. (2) Home for Invalid Ladies, St. Mary's, Dean Park, Bournemouth. Payment £1 1s. per week, or sharing bedroom with another, 17s. 6d. (3) All Saints' Convalescent Home, 8, Pevensey Road, St. Leonards. Apply Sisters of All Saints, Margaret Street, London. Terms 7s. 6d. a week. Private room extra. (4) St. Raphael's Convalescent Home, Higher Lincombe Road, Torquay. Payment 10s. a week. Invalid ladies taken at a higher rate. We hope one or another of these may suit Nurse H. Martin.

*Compensatory Monthly Fees.*

(126) I was engaged to nurse a confinement case which did not take place until exactly a month after the time appointed. I had to hold myself in readiness daily, and could not take other work. I was living in lodgings at my own expense, and my patient now refuses to pay me any compensation for the month I was kept waiting. Can I legally claim full fees or half fees? I nursed her for three weeks, and during that time I was telegraphed for, and had to refuse a good case. Surely I am justified in asking some compensation.—*Sister Lena.*

Would you kindly inform me at your earliest convenience if I can sue for half fees for a monthly case I had booked for June, and which has already taken place? I have written to the lady asking her, as I believe that is the usual thing. I have three letters making the engagement.—*Nurse Roche.*

Will the Editor kindly give the law in the following case? A nurse was engaged for February about the beginning of October. On November 11th the patient expressed by letter some surprise at not having seen the nurse, and said she had forgotten at the interview to say that she expected some washing done, as all her friends' nurses did it. Nurse replied on November 12th, reminding the patient that nothing had been said about it, and declining to do this work. No acknowledgment of this letter was made. When the nurse called on the patient on December 28th she found her not at home, and a letter was received by the last post saying that Mrs —— had engaged someone else, as she could not engage anyone who would not do washing. She asked also why the nurse wanted to see her, and declined to receive her. The nurse has refused two cases for other doctors in consequence of this engagement. Can she claim any recompense?—*Barnet.*

Here are three letters all upon the subject of claiming compensation fees by monthly nurses, and the answer to one and all is that before any legal claim to compensation can be laid, proof of engagement must be made in writing. As Nurse Roche has the letter engaging, she could claim half fees later on, but not before the expiration of the first three months; but although Nurse Lena has a moral right to half fees, she cannot legally claim them, as she has not her engagement in writing; whilst "Barnet," in our opinion, refused her case when she declined to do the washing, as it is still customary for some nurses to do a little for the baby. If she had not intended to give up the case, she ought to have called at once—not allowing nearly six weeks to elapse first—on her patient, and made definite arrangements. We have taken the best advice on this matter for our correspondents, and we know that most of these troubles would be avoided if they belonged to an association like the Midwives' Institute (12, Buckingham Street, Strand), as they would then be given a form for their prospective patients to sign which provides against all these emergencies, and upon which they could legally recover compensation.

*Monthly Work,*

(127) Will "Elizabeth" kindly send her name for our own satisfaction, and not for publication?

### ANSWERS REQUESTED.

*Male Nurse.*

(128) Can anyone kindly give me any information as to the best way for a man to qualify as a male nurse, the names of institutions, &c.?—*Gretna.*

